# HYSPAIN CLINICAL TRIAL Testing the Efficacy of Paracervical Anesthesia for Pain Control During Office Hysteroscopy: A Randomized, Single-Center, Double-Blind, Placebo-Controlled Clinical Trial

**DOI:** 10.3390/jcm13247856

**Published:** 2024-12-23

**Authors:** María Adrien-Lara, Augusto Pereira, Salvatore Giovanni Vitale, Mar Ríos, Tirso Pérez-Medina, Laura Calles-Sastre

**Affiliations:** 1 Gynaecology and Obstetrics Department, Hospital Universitario Puerta de Hierro, Majadahonda, 28222 Madrid, Spain; augusto.pereira@salud.madrid.org (A.P.); mariamar.rios@salud.madrid.org (M.R.); tirso.perez@salud.madrid.org (T.P.-M.); laurabeatriz.calles@salud.madrid.org (L.C.-S.); 2 Division of Gynecology and Obstetrics, Department of Surgical Sciences, University of Cagliari, 09124 Cagliari, Italy; salvatoreg.vitale@unica.it

**Keywords:** office hysteroscopy, pain, paracervical anesthesia, VAS

## Abstract

**Introduction:** Hysteroscopy is a key gynecological procedure for diagnosing and treating endometrial conditions. While hysteroscopy is often performed in office settings without sedation, patients frequently report significant pain during the procedure. This study aims to evaluate the efficacy of paracervical anesthesia with mepivacaine compared to placebo in managing pain during office hysteroscopy. **Methods:** This randomized, single-center, double-blind, placebo-controlled trial was conducted at Puerta de Hierro University Hospital (Madrid, Spain) from June 2021 to June 2022. A total of 108 women were randomized to receive either mepivacaine 2% or a saline placebo prior to hysteroscopy. Pain was assessed using a visual analog scale (VAS) at various stages of the procedure. **Results:** The results showed no significant differences in pain levels between the mepivacaine and placebo groups during the procedure. Both groups exhibited similar rates of complications and procedural difficulties. Factors influencing perceived pain included a history of vaginal delivery and the type of instruments used, but anesthesia did not demonstrate a significant impact on pain reduction in any subgroup. **Discussion**: Our findings indicate that paracervical anesthesia with mepivacaine does not significantly reduce pain during office hysteroscopy, consistent with previous studies. The variability in pain experiences suggests that individual factors, such as pain thresholds and anxiety, may play a significant role. A more personalized approach to pain management, combining pharmacological and non-pharmacological strategies, may be necessary to enhance patient comfort. Future research should involve larger multicenter trials to further explore these findings.

## 1. Introduction

Hysteroscopy, a gynecological endoscopic technique, involves visualizing the vagina, endocervical canal, and endometrial cavity using an optical system. Hysteroscopy is now a cornerstone for diagnosing and treating endocavitary pathology, often regarded as the “gold standard” for diagnosing conditions such as endometrial cancer, endometrial hyperplasia, polyps, and submucosal fibroids [1,2]. In the United States, its use is increasing due to its high efficacy, safety, and patient satisfaction. It is estimated that thousands of hysteroscopies are performed annually in the United States, though exact publicly available figures have not been provided [3].

Currently, most hysteroscopic procedures are conducted in office settings without sedation or general anesthesia. This approach facilitates easy recovery for patients, allowing them to resume normal activities promptly and avoiding the risks associated with surgery and general anesthesia. Office hysteroscopy is considered cost-effective because it avoids more invasive procedures that require operating rooms and general anesthesia, thereby reducing overall healthcare costs.

Several studies have been published that examine the tolerability of office-based hysteroscopy. One such study [4], which includes 1114 patients undergoing hysteroscopy without adjuvant treatments, concludes that despite the procedure being performed atraumatically by expert hysteroscopists, up to 34.8% of patients experience severe pain regardless of age or other risk factors such as nulliparity or cervical canal abnormalities. In this context, multiple lines of research have been developed, seeking both technical and therapeutic alternatives aimed at minimizing pain and discomfort in these patients.

Pain during hysteroscopy is frequently associated with the process of dilating or entering the cervical canal. The cervix has a rich innervation primarily from the pelvic splanchnic nerves (parasympathetic) and hypogastric plexus (sympathetic). When these nerves are stimulated by mechanical distention, such as with cervical dilation during hysteroscopy, they can trigger pain signals. Additionally, the procedure may cause transient ischemia due to the compression of blood vessels within the cervical tissue, contributing to pain experience.

In cases where no dilation is needed, pain can still arise due to the manipulation of the endometrium or the insertion of the hysteroscope. The distention of the uterine cavity with saline or carbon dioxide, used for better visualization, can stretch the endometrial lining and myometrium, which also contain sensory fibers, leading to discomfort.

Moreover, pre-existing conditions play a significant role in the perception of pain during hysteroscopy. Women with cervical stenosis, endometriosis, or pelvic inflammatory disease may experience increased sensitivity or have altered anatomical structures, making them more susceptible to pain during the procedure.

Regarding the technical aspects of hysteroscopy, evidence suggests that avoiding the use of the speculum and cervical traction with a tenaculum significantly reduces procedure-related pain and enhances patient tolerability [5]. Vaginoscopy is faster to perform, less painful, and has a higher success rate, making it the preferred technique for women undergoing office hysteroscopy. However, despite the benefits of the vaginoscopic approach, many patients continue to experience pain during the procedure, with pain being the main reason for not being able to complete it.

Several studies have explored the potential benefits of administering local anesthetics immediately prior to the procedure. The existing body of research, however, is varied in terms of the drugs used, dosages, routes of administration, and observed outcomes.

In the published placebo-controlled clinical trials, drugs have been administered intracavitarily [5] or via the endocervical canal [6,7,8,9] or injected paracervically [10,11] or at the cervical level [12,13]. Other clinical trials have tested anesthetic gels applied to the ectocervical and endocervical areas or as a spray [14,15]. The most commonly used anesthetics in these studies have been lidocaine [6,7,8,11,12,13], mepivacaine [5,10], and prilocaine [9], with dosages ranging between 3 and 10 mL depending on the study.

Based on the findings from these clinical trials involving local anesthesia in various forms, most studies demonstrate some degree of pain reduction during the procedure. However, a key concern raised is the clinical significance of this pain relief, as the difference in visual analog scale (VAS) scores between the treatment and control groups is minimal.

Another limitation found in the literature is that many authors fail to consider other risk factors that may heighten patients’ pain perception during hysteroscopy.

This study aimed to evaluate the efficacy of paracervical anesthesia with mepivacaine for pain control during office hysteroscopy compared to placebo. Additionally, the objective was to investigate the specific advantages of administering local anesthesia in particular patient groups with associated risk factors.

## 2. Materials and Methods

This randomized, single-center, double-blind, placebo-controlled clinical trial was conducted between June 2021 and June 2022 at the Department of Gynecology of Puerta de Hierro University Hospital, Majadahonda, Madrid, Spain. This study received approval from the AEMPS (Spanish Agency for Medicines and Health Products) and the hospital’s ethical committee on 12 April 2021. Written informed consent was obtained from all participants. This study was also registered in European Clinical Trial Database (EudraCT number 2021-001182-21).

Inclusion criteria encompassed women over 18 years old with an indication for office hysteroscopy, while exclusion criteria included a history of allergy or intolerance to local anesthetics, contraindications to or inability to cooperate during office hysteroscopy, chronic pain that requires daily analgesic medication or deep endometriosis history, Essure^®^ device carriers, need for procedures involving hysteroscopic morcellators, and inability to comprehend this study’s nature and provide written informed consent. A CONSORT flow diagram was used to document the flow of participants through the different stages of this study [16].

The sample size was calculated based on the expected difference in pain scores between the two groups. Assuming a mean global pain score on the visual analog scale (VAS) of 5 in the placebo group and 3.5 in the anesthesia group, with a standard deviation of 2.5 in both groups, we determined that 45 patients per group would be necessary to achieve sufficient statistical power. This calculation was based on an 80% power and a significance level of 0.05. Given an expected dropout rate of 15%, we estimated that 54 patients per group needed to be recruited, totaling 108 patients for this study.

A total of 108 eligible women were randomized into two groups. A random allocation sequence was generated by an independent statistician using computer-generated random numbers. The allocation was concealed using sequentially numbered, opaque, sealed envelopes. Participants, clinicians, and outcome assessors were blinded to group assignments.

One group received placebo with physiological saline solution, while the other received mepivacaine 2%. Both substances were administered locally with a spinal needle (Quincke needle tip 22Gx3.00”—Becton Dickinson S.A.) in the posterior vaginal fornix at 5 and 7 o’clock, with 5 mL on each side. The nurse responsible for the hysteroscopy clinic prepared the medication assigned to the patient in an adjacent room. The syringes used in both cases were identical, and the contents of the vials were indistinguishable to the naked eye, as both contained a colorless liquid.

Additionally, all patients received premedication orally with 5 mg diazepam, 600 mg ibuprofen, and 10 mg butylscopolamine, as used in usual clinical practice. Office hysteroscopy was performed according to standard clinical practice conducted by the same specialist. Hysteroscopy was performed using a continuous-flow hysteroscope (Bettocchi). The optical system was 2.9 mm in size, and the working channel was 5 Fr in size. For the procedure, the chosen distension medium was saline solution, using a mechanical pump to control the instillation flow and intrauterine pressure.

Pain was assessed using a visual analog scale (VAS) for pain scored from 1 to 10 during the paracervical administration of mepivacaine or placebo [17], after the first access to the uterine cavity, and just after finishing the procedure to evaluate overall pain. We recorded age, race, parity, previous cesarean section, menopause, previous dilation and curettage, previous conization, previous tamoxifen treatment, previous chemo- or radiotherapy, and indication for the procedure. Procedure data included hysteroscopic findings, kind of procedure, instruments used, and complications. We recorded the procedure time from the first access to the endometrial cavity until the procedure was completed. The mean flow pressure was also recorded.

Statistical analysis followed the principles specified in the International Conference on Harmonization [18] using Stata/IC version 16. A description of baseline and sociodemographic characteristics was provided for each group. Data were represented as absolute frequencies and percentages for qualitative variables. For quantitative variables, the mean and standard deviation (SD) or the median and interquartile range (IQR: P25; P75) were used, depending on the distribution. For ordinal variables, absolute frequencies and percentages or the median and IQR were used, depending on the number of categories.

For the analysis of the primary variable, a non-parametric test was conducted using the Mann–Whitney U test. The same Mann–Whitney U test was applied for the secondary variables. The level of statistical significance was set at 0.05.

A multivariate analysis was conducted using multiple linear regression to assess the influence of several variables on the overall pain reported during the procedure. Initially, all variables of interest were included in the model, and a backward modeling strategy was employed to identify variables that significantly predicted pain. Variables with a *p*-value < 0.05 were retained in the model.

For safety analysis, the adverse effects observed in each group were described using absolute and relative frequencies, along with their 95% confidence intervals.

## 3. Results

**Figure 1:**
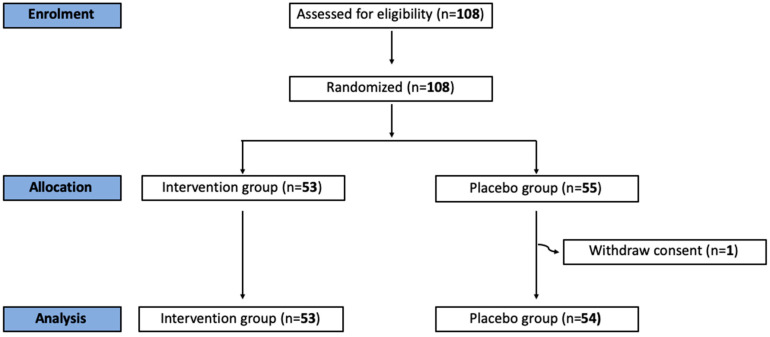
Flowchart of recruited patients.

A total of 108 women with an indication for office hysteroscopy were included and randomized in this study. One participant withdrew consent prior to treatment administration and was subsequently excluded. A CONSORT flow diagram is shown in Figure 1.

The demographic and clinical characteristics of the recruited patients are presented in Table 1. It can be observed that the characteristics of both groups are comparable, with no significant differences identified.

As shown in Table 2, there were no significant differences in the procedures performed. Both groups exhibited a similar rate of stenotic or tortuous accesses, with only one case of failed access in the treatment group. The mean procedure time was similar in both groups. However, there was a significant variation in duration between procedures, ranging from 2 to 60 min in both cases. This is because some procedures were purely diagnostic, involving a simple “in-and-out” approach, and were therefore very short, while others were surgical, requiring additional interventions. Moreover, the findings and procedures conducted in both groups were largely comparable, with the exception of a few procedures, such as septoplasty and isthmoplasty, which were performed infrequently and showed minimal significance. Complications were also comparable in both groups, with one failed hysteroscopy in each group and a low rate of cavity distension failure and abnormal uterine bleeding.

The results of the main variable analysis are presented in Table 3. The average global pain during the procedure, as well as pain during the first entry of the hysteroscope into the endometrial cavity and pain resulting from mepivacaine infiltration, was similar in both groups, with no significant differences observed in reported pain between the two groups. We observed no significant differences in perceived pain during paracervical infiltration with mepivacaine compared to placebo. In both groups, the pain reported was mild, with a median pain score of 3.

The association between secondary variables and patient-perceived pain was also assessed, as shown in Table 4. The factors that significantly reduced pain during hysteroscopy included a history of vaginal delivery and the use of mechanical instruments compared to bipolar instruments. We did not find any significant differences in perceived pain among patients in relation to hysteroscopy duration, the mean pressure during the procedure, or other personal medical history such as menopause, previous conization, curettage, cesarean section, or tamoxifen treatment. However, when evaluating the effect of anesthesia in these patient subgroups, no statistically significant differences in pain reduction were found.

Multivariate analysis revealed that no variable was statistically significantly associated with pain, either globally or during the first entry into the uterine cavity.

No significant adverse events were reported during this study.

## 4. Discussion

This study aimed to evaluate the efficacy of paracervical anesthesia with mepivacaine for pain control during office hysteroscopy compared to placebo. The results obtained showed no significant differences in the pain experienced by patients between the intervention group and the control group, both during the initial insertion of the hysteroscope into the uterine cavity and throughout the procedure.

Our findings align with those of Costello et al. [7], who also found that local anesthetic injected through the hysteroscope did not significantly reduce pain during outpatient hysteroscopy and endometrial biopsy compared to placebo. Similarly, Lau et al. [8] reported no significant benefits of paracervical anesthesia in outpatient hysteroscopy settings. These consistent findings suggest that local anesthetics, such as mepivacaine, may not be effective in reducing pain for all patients undergoing hysteroscopy.

Topical anesthesia has also been explored with heterogeneous results. Cicinelli et al. [6] found that topical anesthesia was effective in reducing pain during diagnostic hysteroscopy and endometrial biopsy in postmenopausal women. Esteve et al. [9] supported these findings, showing that intracervical lidocaine reduced pain during outpatient hysteroscopy. However, Wong et al. [13] reported that stepwise pain score analysis showed no significant effects of local lignocaine in reducing pain in outpatient hysteroscopy.

It is noteworthy that the minimal difference in pain levels and the low overall pain reported may be related to the fact that all patients were premedicated with ibuprofen, diazepam, and butylscopolamine, which may have influenced the effects of the anesthesia.

It is essential to recognize that the pain experienced during hysteroscopy is influenced by a complex interplay of factors beyond cervical dilation, including uterine distention, the activation of nociceptors, and individual anatomical variations. The cervix is richly innervated by the pelvic splanchnic nerves and the hypogastric plexus, which can trigger pain signals when subjected to mechanical distention or manipulation. Conditions such as cervical stenosis and uterine fibroids can complicate the procedure, increasing the likelihood of discomfort. The variability in pain management outcomes further emphasizes the importance of individual factors, such as pain thresholds and anxiety levels. Therefore, a personalized approach to pain management, possibly combining pharmacological and non-pharmacological methods, may be necessary to enhance patient experiences.

Our study has several limitations. The sample size, while adequate, may not capture all variations in pain perception and response to anesthesia. Additionally, this study was conducted at a single center, which may limit the generalizability of the findings. Future research should focus on larger multicenter trials to validate these results and explore the efficacy of combined pain management strategies.

In summary, this study contributes to the accumulating evidence indicating that paracervical anesthesia with mepivacaine does not significantly alleviate pain during office hysteroscopy. Our results mirror those of previous studies, which similarly found no substantial pain reduction with local anesthetics in this context. The variability in pain management outcomes suggests that individual factors—such as pain thresholds and anxiety—may play a crucial role. Given these findings, a more personalized approach to pain management, potentially integrating both pharmacological and non-pharmacological strategies, may be necessary to improve patient comfort during hysteroscopy. While our study has limitations, including its sample size and single-center design, further multicenter research is warranted to validate these findings and investigate the effectiveness of combined pain management approaches.

## Figures and Tables

**Table 1:** Baseline data: D&C = dilation and curettage; TMX: tamoxifen; QRT: chemo- or radiotherapy; US: ultrasound. Age expressed as median (p25–p75).

Characteristic	Anaesthesia (*n* = 53)	Placebo (*n* = 54)
Age (y)	44y (31–74)	44.5y (31–72)
Race		
Caucasian	42 (79.25%)	43 (79.44%)
Hispanoamerican	10 (18.87%)	11 (20.37%)
Interracial	1 (1.89%)	0
Parity		
Nulliparous	21 (39.62%)	32 (38.89%)
Primiparous	14 (26.42%)	9 (16.67%)
Multiparous	18 (33.96%)	24 (44.44%)
Previous Cesarean section	10 (18.87%)	16 (29.63%)
Menopause	15 (28.30%)	15 (27.78%)
Previous D&C	9 (16.98%)	11 (20.37%)
Previous Conization	4 (7.55%)	3 (5.56%)
Previous TMX treatment	5 (9.43%)	3 (5.56%)
Previous QRT	4 (7.55%)	1 (1.85%)
Indication		
Infertility	9 (16.98%)	9 (16.67%)
Us finding	19 (35.85%)	18 (33.33%)
Abnormal uterine bleading	16 (30.19%)	16 (29.63%)
Postmenopause haemorrage	8 (35.85%)	8 (14.81%)
Other	1 (15.09%)	3 (5.56%)

**Table 2:** Hysteroscopic findings and procedures.

Hysteroscopyc Findings and Procedures	Anaesthesia (*n* = 53)	Placebo (*n* = 54)
Entrance		
Normal–easy	41 (77.36%)	39 (72.22%)
Stenotic	5 (9.43%)	5 (9.26%)
Tortuous	6 (11.32%)	10 (18.52%)
Failed	1 (1.89%)	0
Endocervical canal		
Normal	45 (84.91%)	49 (90.74%)
Pathology	8 (15.09%)	5 (9.26%)
Uterine cavity findings	38 (73.08%)	38 (70.37%)
Polyp	26 (50%)	28 (51.85%)
Myoma	8 (15.38%)	7 (12.96%)
RPOC (retained products of conception)	5 (9.62%)	3 (5.56%)
Procedure		
Diagnostic	11 (21.15%)	14 (26.42%)
Therapeutic	41 (78.85%)	39 (73.58%)
Polypectomy	26 (49.06%)	30 (55.56%)
Myomectomy	7 (13.21%)	7 (12.96%)
Septoplasty/metroplasty	1 (1.89%)	0
Isthmoplasty	2 (3.77%)	0
RPOC resection	5 (9.43%)	3 (5.56%)
Instruments used		
Bipolar	37 (74%)	38 (76%)
Mechanical	13 (26%)	12 (24%)
Complications		
Failed hysteroscopy	1 (1.89%)	1 (1.85%)
Insuficient cavity distension	1 (1.89%)	2 (3.70%)
Bleeding	1 (1.89%)	0
Mean procedure time (min)	10 (2–60)	11 (2–60)
Mean flow pressure (mmHg)	75 (65–150)	75 (65–135)

**Table 3:** Main variable results.

	Anaesthesia	Placebo	*p*
Global pain	4 (2–6)	4 (3–6)	0.5822
Pain during entrance	3 (1–6)	3 (1–6)	0.3948
Pain related to infiltration of Mepivacaine	3 (1–4)	3 (1–4)	0.4650
EVA: Median (p25–p75)			

**Table 4:** Association between secondary variables and overall pain adjusted by treatment group.

	Coefficient (IC 95%)	*p*	Treatment Coefficient (Placebo)
Age	−0.00 (−0.05; 0.03)	0.717	0.39 (−0.49; 1.28)
Ethnicity			
Caucasian	REF. CAT.		
Hispanoamerican	0.52 (−0.56; 1.62)	0.343	0.48 (−0.38; 1.35)
Other	5.35 (0.8; 9.89)	0.021	
Vaginal deliveries			
0	REF. CAT.		
>=1	−1.14 (−2.02; −0.26)	0.012	0.40 (−0.46; 1.26)
Cesarean section	0.39 (−0.64; 1.43)	0.454	0.34 (−0.54; 1.24)
D&C (dilatation and curettage)			
Conization	0.13 (−1.6; 1.93)	0.882	0.39 (−0.49; 1.28)
Menopause	0.49 (−0.49; 1.47)	0.323	0.39 (−0.49; 1.28)
Treatment with Tamoxifen	−0.94 (−2.63; 0.74)	0.268	0.35 (−0.53; 1.24)
Duration of hysteroscopy	0.023 (−0.02; 0.06)	0.290	0.32 (−0.56; 1.2)
Mean pressure	0.00 (−0.01; 0.02)	0.664	0.33 (−0.55; 1.22)
Instruments used			
Bipolar	REF. CAT.		
Mechanical	−1.11 (−2.10; −0.11)	0.029	0.33 (−0.52; 1.2)

## Data Availability

The raw data supporting the conclusions of this article will be made available by the authors on request.
